# Risk of acute kidney injury associated with the albumin–bilirubin score in ICU patients with acute pancreatitis: A cohort study

**DOI:** 10.1097/MD.0000000000050677

**Published:** 2026-09-11

**Authors:** Yin-ying Wang, Hui-bo Wang

**Affiliations:** a Department of Infectious Disease, Beijing Luhe Hospital, Capital Medical University, Beijing, China; b Hebei Medical University, Shijiazhuang, Hebei, China; c Department of General Surgery, Handan Central Hospital, Handan, Hebei, China.

**Keywords:** acute kidney injury, acute pancreatitis, ALBI score, critically ill patients, MIMIC-IV database

## Abstract

Acute kidney injury (AKI) is a frequent and clinically significant complication in patients with acute pancreatitis (AP), and early recognition is essential for optimal management. This study investigated the association between the albumin–bilirubin (ALBI) score and the development of AKI in critically ill patients with AP. Clinical data for first-time intensive care unit (ICU) admissions with a confirmed AP diagnosis were retrospectively extracted from the Medical Information Mart for Intensive Care IV database. Total bilirubin and albumin were collected within the first 24 hours of ICU admission. Multivariable logistic regression models were used to assess the association between ALBI score (as continuous, binary, and tertile variables) and AKI risk, adjusting for confounders including demographics, comorbidities, and laboratory values. A total of 529 ICU patients with AP were included, with an AKI incidence of 69.4%. Multivariable analyses demonstrated a significant linear association between a higher ALBI score and increased risk of AKI. These findings remained consistent across subgroup and sensitivity analyses. After adjusting for potential confounders, each 1-unit increase in ALBI score was associated with a 109% increase in the risk of AKI (odds ratio [OR] = 2.09; 95% confidence interval [CI] = 1.39–3.15; *P* < .001). When categorized, patients in the highest ALBI groups had significantly higher risk compared to the reference groups (grade 3: OR = 1.98; 95% CI = 1.24–3.17, *P* = .004; T3: OR = 2.18; 95% CI = 1.24–3.84, *P* = .007). Upon admission, a higher ALBI score is independently associated with AKI in ICU patients with AP. These results underscore the potential of the ALBI score as a valuable tool for early risk stratification in critically ill patients with AP. Multicenter and larger sample sizes are needed to validate these findings.

## 1. Introduction

Acute pancreatitis (AP) remains a prevalent gastrointestinal emergency, with approximately 20% to 25% of cases progressing to severe forms characterized by organ failure^[1]^ and mortality rates reaching 30%.^[2]^ Acute kidney injury (AKI) is one of the most common and severe complications in AP,^[3]^ affecting 14% to 43% of patients. AKI in the context of AP is not merely a marker of dysfunction but a primary driver of prolonged hospitalization, increased costs, and mortality. Despite improvements in supportive care, early identification of AKI remains a clinical challenge, necessitating the search for accessible and reliable markers for risk stratification.

The albumin–bilirubin (ALBI) score, calculated from serum albumin and total bilirubin (TBIL), was originally designed as an objective tool for liver function assessment in oncology.^[4]^ However, its utility has recently expanded to broader critical care contexts, showing promise in predicting outcomes for sepsis, chemotherapy-induced AKI,^[5]^ and the evaluation of acute upper gastrointestinal bleeding.^[6]^ While previous research indicates that the ALBI score correlates with in-hospital mortality in patients with AP^[7]^ and an elevated ALBI grade has been associated with adverse outcomes in patients with AKI^[8]^ in the intensive care unit (ICU), its specific relationship with renal complications in this population has not been established. Given that both albumin (reflecting inflammatory state and capillary leak) and bilirubin (linked to oxidative stress and bile acid-induced tubular toxicity) are integral to AP pathophysiology, the ALBI score may offer a composite reflection of the risk environment leading to kidney injury.

Accordingly, we hypothesized that the ALBI score is independently associated with the development of AKI in patients with AP. Using data from the Medical Information Mart for Intensive Care IV (MIMIC-IV) database, this study aimed to evaluate whether the ALBI score can serve as a clinical indicator for identifying AP patients at elevated risk for AKI.

## 2. Materials and methods

### 2.1. Study design and population

The data for this study were extracted from version 3.1 of the MIMIC-IV database, a publicly available repository developed and maintained by the Massachusetts Institute of Technology Computational Physiology Laboratory (https://physionet.org/content/mimiciv/3.1/). This database contains anonymous medical records for 94,458 adult ICU patients treated at Beth Israel Deaconess Medical Center from 2008 to 2022. The first author obtained access to the database (certification number: 60,756,767 and 65,444,380) after completing the required certification and signing a data use agreement. The Institutional Review Boards of the Massachusetts Institute of Technology and the Beth Israel Deaconess Medical Center granted ethical clearance for retrospective analysis of the de-identified dataset. Considering the retrospective nature and anonymized characteristics of the dataset, approval for waiving informed consent was obtained. The investigation complies with the Strengthening the Reporting of Observational Studies in Epidemiology guidelines for reporting observational studies in epidemiology.^[9]^

**Figure 1.**
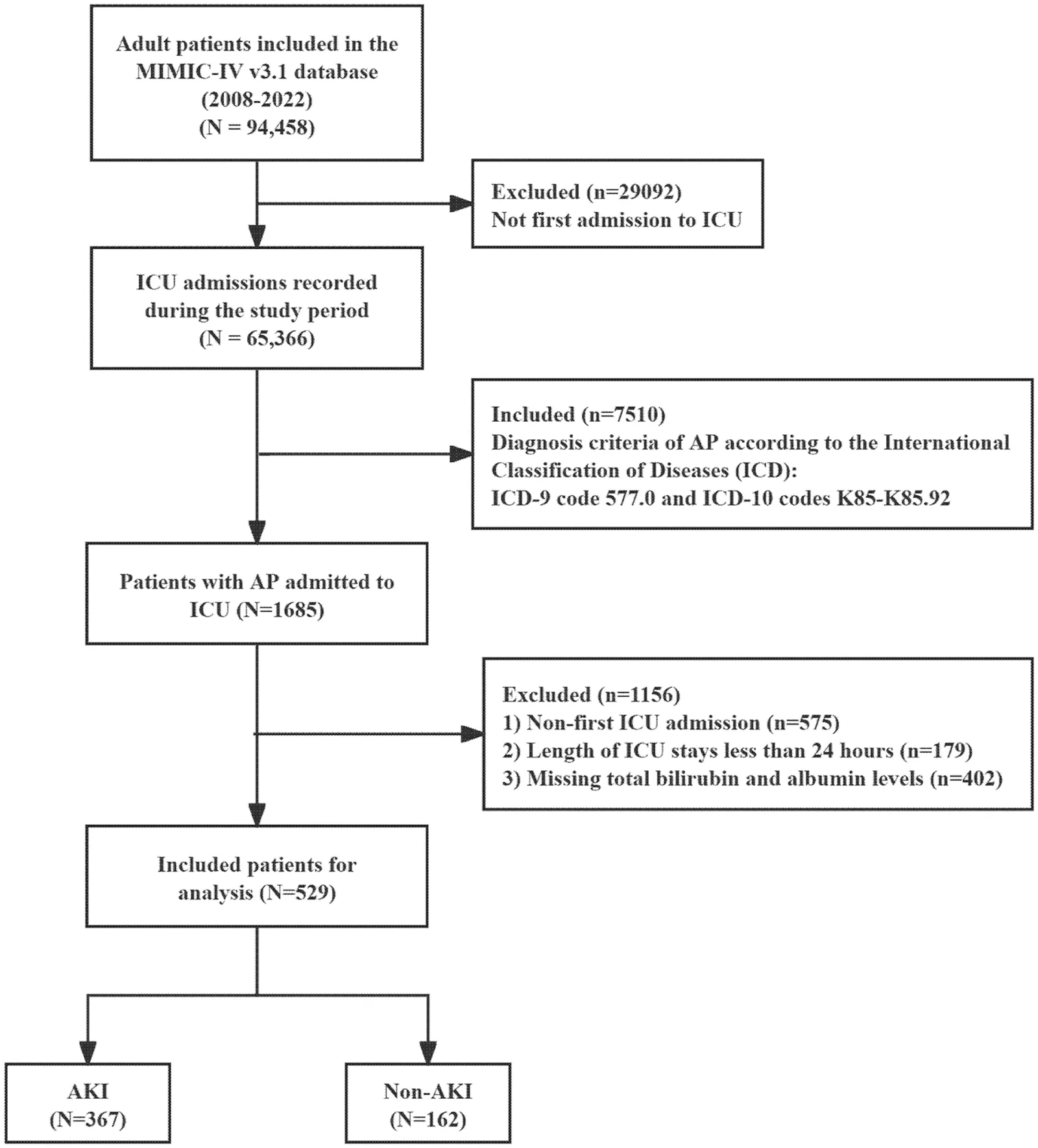
Flowchart of inclusion and exclusion criteria. AKI = acute kidney injury, AP = acute pancreatitis, MIMIC = Medical Information Mart for Intensive Care, ICU = intensive care unit.

An adult patient cohort aged over 18 years was extracted from the database. AP was diagnosed according to the International Classification of Diseases (ICD) criteria, specifically using ICD-9 code 577.0 and ICD-10 codes K85-K85.92. A total of 7510 patients with AP were included in the study, of whom 1685 were admitted to the ICU. The exclusion criteria were as follows: non-first ICU admissions (n = 575); ICU stays shorter than 24 hours (n = 179); missing bilirubin or albumin levels on the first day of ICU admission (n = 402). Ultimately, 529 patients were enrolled in this study (Fig. 1).

### 2.2. Variable extraction and outcome

Data extraction was performed using PostgreSQL (version 13.6.1; PostgreSQL Global Development Group) and Navicat Premium (version 17; PremiumSoft CyberTech Limited) via SQL queries.

ALBI score was the exposure variable. Initial TBIL and albumin levels during the initial 24-hour period following admission to the ICU were analyzed. The calculation formula for the ALBI score is (log_10_ TBIL [μmol/L] × 0.66) + (albumin [g/L] × [−0.085]). Included patients were stratified according to the standard ALBI grading system into grade 3 (ALBI score > −1.39) and non-grade 3 groups (ALBI score ≤ −1.39). Subsequently, participants were stratified into tertiles (T1: ≤ −1.79, T2: −1.79 to −1.29, and T3: >−1.29) based on ALBI score distributions. The non-grade 3 and T1 subgroups were designated as the reference group, respectively.

The outcome focused on AKI in critically ill patients with AP. AKI was defined according to the Kidney Disease: Improving Global Outcomes (KDIGO) criteria,^[10]^ which include any of the following: an increase in serum creatinine (Scr) ≥0.3 mg/dL (≥26.5 µmol/L) within 48 hours; Scr levels ≥1.5 times baseline value within 7 days; or urine volume <0.5 mL/kg/h for ≥6 consecutive hours.

The extracted variables included demographic characteristics, vital signs, comorbidities, laboratory tests, disease severity, intervention measures, and clinical outcomes. Baseline variables were obtained within the first 24 hours after ICU admission (Table 1). Potential confounders were selected based on existing literature and clinical judgment. Multicollinearity^[11]^ was assessed using variance inflation factors, with values <5 indicating no significant multicollinearity.

**Table 1:** Baseline characteristics and outcome of ICU patients with AP, categorized by ALBI score tertiles.

Variables	Total (n = 529)	ALBI tertiles			*P* value
		T1: ≤−1.79 (n = 175)	T2: −1.79 to −1.29 (n = 177)	T3: >−1.29 (n = 177)	
Demographics					
Age (yr)	58.2 ± 17.9	56.4 ± 17.5	60.7 ± 18.7	57.4 ± 17.2	.055
Sex, male, n (%)	294 (55.6)	95 (54.3)	97 (54.8)	102 (57.6)	.794
Race, White, n (%)	325 (61.4)	110 (62.9)	110 (62.1)	105 (59.3)	.771
Vital signs					
Heart rate (beats/min)	97.7 ± 18.2	97.7 ± 18.5	97.6 ± 18.7	97.8 ± 17.5	.996
MBP (mm Hg)	81.6 ± 13.4	85.4 ± 14.2	81.8 ± 13.6	77.6 ± 11.0	<.001
Respiratory rate (breaths/min)	21.6 ± 4.4	21.3 ± 4.4	21.5 ± 4.2	22.1 ± 4.6	.245
Temperature (°C)	37.0 ± 0.7	37.1 ± 0.6	37.1 ± 0.7	36.9 ± 0.6	.01
Laboratory tests					
AG (mmol/L)	16.4 ± 5.6	17.1 ± 5.2	15.9 ± 5.2	16.2 ± 6.3	.104
BUN (mg/dL)	22.0 (12.0–38.0)	20.0 (11.0–34.0)	21.5 (12.8–35.0)	24.0 (15.0–45.0)	.025
Calcium (mmol/L)	7.8 ± 1.2	8.2 ± 1.3	7.7 ± 1.2	7.5 ± 1.1	<.001
Creatinine (mg/dL)	1.1 (0.7–2.2)	1.0 (0.7–1.7)	1.1 (0.8–2.1)	1.4 (0.7–2.7)	.137
Potassium (mmol/L)	4.2 ± 0.9	4.2 ± 1.0	4.1 ± 0.7	4.2 ± 0.9	.699
Sodium (mmol/L)	138.3 ± 6.3	139.7 ± 6.1	138.7 ± 5.7	136.5 ± 6.7	<.001
ALBI (score)	−1.5 (−1.9 to −1.1)	−2.1 (−2.3 to −1.9)	−1.6 (−1.7 to −1.4)	−0.9 (−1.1 to −0.7)	<.001
Albumin (g/dL)	2.9 ± 0.6	3.4 ± 0.5	2.9 ± 0.4	2.3 ± 0.4	<.001
Total bilirubin (mg/dL)	1.1 (0.6–3.4)	0.7 (0.4–1.1)	1.2 (0.6–3.4)	2.8 (0.9–8.6)	<.001
White blood cell (×10^12^/L)	14.9 ± 8.7	14.1 ± 8.6	14.5 ± 8.4	16.1 ± 9.0	.079
Hemoglobin (g/dL)	11.2 ± 2.5	11.9 ± 2.5	11.2 ± 2.5	10.5 ± 2.4	<.001
RDW (%)	15.3 ± 2.3	14.8 ± 1.8	15.1 ± 2.2	16.0 ± 2.7	<.001
Platelet (×10^12^/L)	211.1 ± 127.3	220.1 ± 118.8	209.2 ± 129.8	204.1 ± 133.0	.491
Comorbidities		17.1 ± 5.2	15.9 ± 5.2	16.2 ± 6.3	.104
CHF, n (%)	75 (14.2)	27 (15.4)	29 (16.4)	19 (10.7)	.265
Chronic kidney disease, n (%)	78 (14.7)	26 (14.9)	28 (15.8)	24 (13.6)	.834
Diabetes, n (%)	168 (31.8)	63 (36)	61 (34.5)	44 (24.9)	.051
End-stage renal disease, n (%)	14 (2.6)	3 (1.7)	6 (3.4)	5 (2.8)	.707
Hypertension, n (%)	323 (61.1)	116 (66.3)	109 (61.6)	98 (55.4)	.108
Liver failure, n (%)	43 (8.1)	7 (4)	11 (6.2)	25 (14.1)	.001
Malignancy, n (%)	42 (7.9)	11 (6.3)	14 (7.9)	17 (9.6)	.515
Obesity, n (%)	106 (20.0)	26 (14.9)	29 (16.4)	18 (10.2)	.21
Disease severity					
Sepsis, n (%)	106 (20.0)	112 (64)	126 (71.2)	152 (85.9)	<.001
SOFA (score)	6.6 ± 4.3	4.9 ± 3.6	6.4 ± 4.2	8.5 ± 4.3	<.001
Intervention measures					
CRRT, n (%)	94 (17.8)	19 (10.9)	28 (15.8)	47 (26.6)	<.001
Vasopressors, n (%)	86 (16.3)	11 (6.3)	25 (14.1)	50 (28.4)	<.001
Outcome					
AKI, n (%)	367 (69.4)	103 (58.9)	124 (70.1)	140 (79.1)	<.001

Data are presented as mean ± SD, medians (IQR), or frequency (percentage).

AG = anion gap, AKI = acute kidney injury, ALBI = albumin–bilirubin, AP = acute pancreatitis, BUN = blood urea nitrogen, CHF = congestive heart failure, CRRT = continuous renal replacement therapy, IQR = interquartile range, ICU = intensive care unit, MBP = mean blood pressure, RDW = red cell distribution width, SD = standard deviation, SOFA = Sequential Organ Failure Assessment.

### 2.3. Statistical analysis

Statistical normality of ALBI score was assessed using the Shapiro–Wilk test. Descriptive analyses were performed with continuous variables expressed as mean ± standard deviation for normally distributed data or median (interquartile range) for nonparametric distributions; categorical variables were reported as absolute frequencies with percentages. Intergroup comparisons employed appropriate statistical tests: the χ^2^ test or Fisher exact test for categorical variables, while continuous variables were analyzed using parametric tests (independent *t* test for two-group comparisons or analysis of variance for multi-group comparisons) or nonparametric alternatives (Mann–Whitney *U* test for two-group analyses and Kruskal–Wallis *H* test for multi-group assessments), contingent upon distributional characteristics confirmed by normality testing.

To assess the association, both unadjusted and multivariable-adjusted models were developed. Covariates in the fully adjusted model included age, sex, race, temperature, mean blood pressure (MBP), white blood cell (WBC), hemoglobin, platelet, creatinine, blood urea nitrogen (BUN), anion gap (AG), hypertension, diabetes, congestive heart failure, sepsis, and vasopressors. A restricted cubic spline with 4 knots was applied to examine potential nonlinear relationships.

Stratified analyses were performed for clinically relevant subgroups, including age strata, sex, race, hypertension, diabetes, and sepsis. Potential effect modification was assessed using likelihood ratio tests for interaction. Mediation analysis followed the Baron–Kenny causal steps and employed bias-corrected bootstrap confidence intervals (CIs; 10,000 iterations). The magnitude of mediation was quantified using the product-of-coefficients approach, and statistical significance was evaluated using the Sobel test. Indirect effects accounting for ≥5% of the total effect were considered biologically meaningful. To assess potential overadjustment bias, sensitivity analyses were conducted by excluding patients with malignancy, liver failure, and end-stage renal disease. In addition, an alternative model IV was used, excluding creatinine, BUN, sepsis, and vasopressors from model III. Multivariate logistic regression and subgroup analysis were conducted accordingly. The robustness of the findings against unmeasured confounding was further evaluated using *E*-value-based sensitivity analyses.

All analyses were performed using R statistical software (version 4.4.2; R Foundation for Statistical Computing) and the FreeStatistics analysis platform (version 2.1; Clinical Scientists Team). Two-tailed tests were utilized, with a significance level set at *P* < .05.

## 3. Results

### 3.1. Baseline demographic and clinical characteristics

Figure 1 illustrates the detailed inclusion and exclusion procedures used in this study. A cohort of 529 ICU patients with AP was included in the study, comprising 235 (44.4%) females and 294 (55.6%) males. The mean age of all participants was 58.2 ± 17.9 years.

Table 1 summarizes the baseline characteristics and outcomes of the patients with AP, categorized by ALBI tertiles (T1: ≤−1.79, T2: −1.79 to −1.29, and T3: >−1.29). The median ALBI score was −1.5, and the incidence of AKI was 69.4%. The average Sequential Organ Failure Assessment (SOFA) score was 6.6, the incidence of sepsis was 20%, and the vasopressor use rate was 16.3%. Compared with the lower group, the elevated group had higher WBC, BUN, TBIL, ALBI score, and SOFA scores with lower MBP, albumin, and hemoglobin. The use of vasopressors and continuous renal replacement therapy, and the incidence of AKI, were higher in the T3 group. The distribution trend among the tertile groups also increased in the same way.

### 3.2. Association between ALBI score and AKI

**Figure 2.**
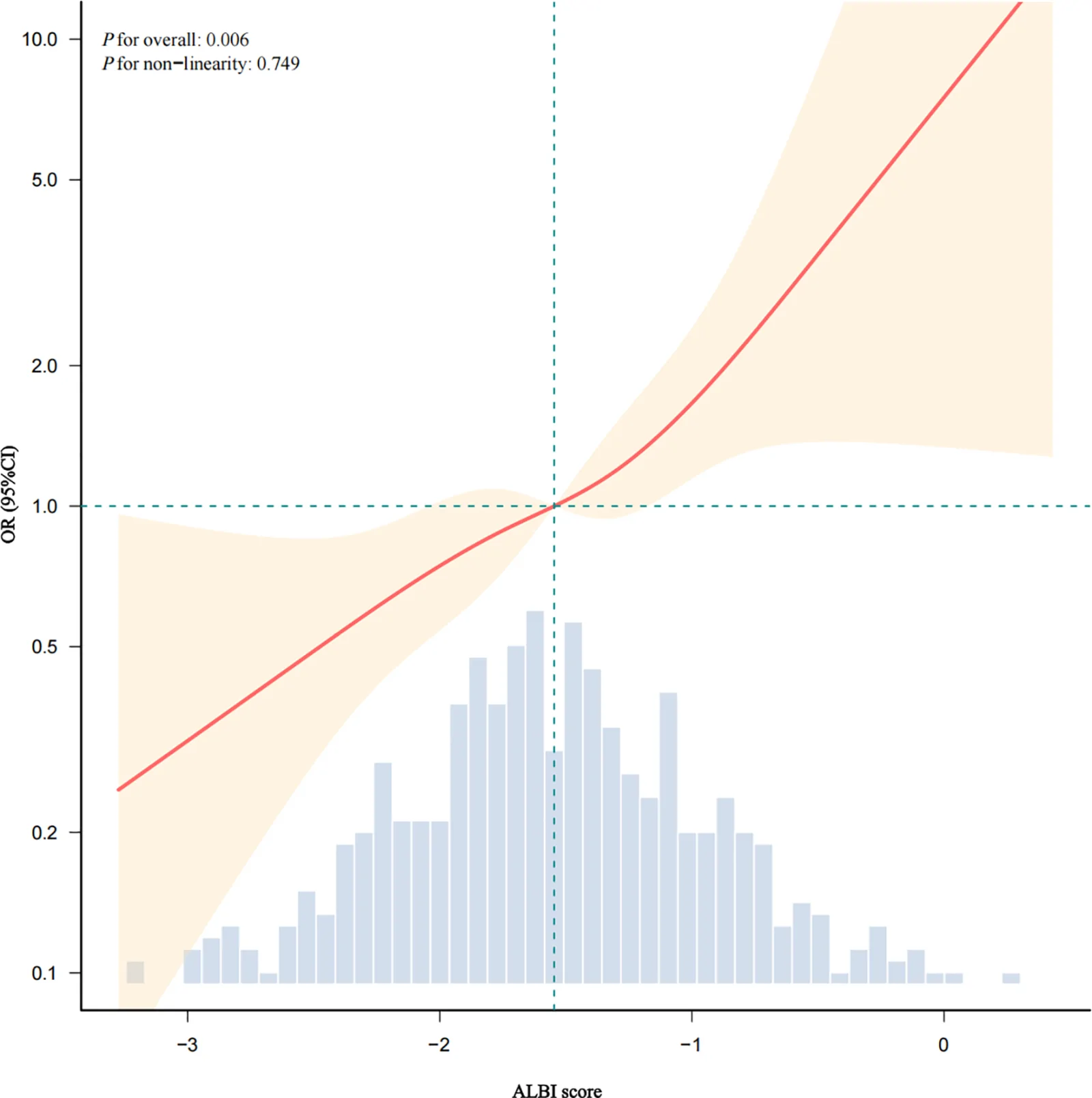
Restricted cubic spline for association of ALBI score and AKI among critically ill patients with AP after all covariate adjustment in model III. AKI = acute kidney injury, ALBI = albumin–bilirubin, AP = acute pancreatitis, CI = confidence interval, OR = odds ratio.

The results of univariate analysis showed that age, MBP, WBC, BUN, creatinine, AG, sepsis, continuous renal replacement therapy, and vasopressors showed a significant association with AKI (*P* < .05). As depicted in the multivariate logistic regression analysis (Table 2), a higher ALBI score was significantly associated with an increased incidence of AKI. When the ALBI score was analyzed as a continuous variable, a 1-unit increase in ALBI score was linked to a 122% higher risk of AKI (odds ratio [OR] = 2.22; 95% CI = 1.60–3.08; *P* < .001) in model I (unadjusted). This association remained significant in model III, which adjusted for potential confounders including age, sex, race, temperature, MBP, WBC, hemoglobin, platelet, creatinine, BUN, AG, hypertension, diabetes, congestive heart failure, sepsis, and vasopressors (adjusted OR = 2.09; 95% CI = 1.39–3.15; *P* < .001). When the ALBI score was categorized into binary and tertiles, adjusted ORs were 1.98 (95% CI = 1.24–3.17; *P* = .004) and 2.18 (95% CI = 1.24–3.84; *P* = .007) for the highest ALBI score group (grade 3, >−1.39; T3, >−1.29) in the fully adjusted model, compared with that of the reference group (non-grade 3, <−1.39; T1, <−1.79; Table 2). Consistent results were obtained across all models, demonstrating the robustness of the statistical results. The restricted cubic spline plot shows a linear correlation between ALBI score and AKI (Fig. 2).

**Table 2:** Multivariate logistic regression analysis of AKI among critically ill patients with AP based on the ALBI score (continuous/binary/tertiles).

Variable	Model I		Model II		Model III	
	OR (95% CI)	*P* value	OR (95% CI)	*P* value	OR (95% CI)	*P* value
Continuous	2.22 (1.60–3.08)	<.001	2.23 (1.60–3.09)	<.001	2.09 (1.39–3.15)	<.001
Binary						
Non-grade 3	1 (Ref)		1 (Ref)		1 (Ref)	
Grade 3	2.19 (1.46–3.28)	<.001	2.29 (1.52–3.44)	<.001	1.98 (1.24–3.17)	.004
Tertiles						
T1	1 (Ref)		1 (Ref)		1 (Ref)	
T2	1.64 (1.05–2.54)	.029	1.58 (1.01–2.47)	.043	1.71 (1.04–2.81)	.034
T3	2.64 (1.65–4.24)	<.001	2.67 (1.66–4.29)	<.001	2.18 (1.24–3.84)	.007
*P* for trend		<.001		<.001		.005

Model I: unadjusted.

Model II: adjusted for age, sex, and race.

Model III: adjusted for age, sex, race, temperature, MBP, WBC, hemoglobin, platelet, AG, hypertension, diabetes, CHF, creatinine, BUN, sepsis, and vasopressors.

AG = anion gap, AKI = acute kidney injury, ALBI = albumin–bilirubin, AP = acute pancreatitis, BUN = blood urea nitrogen, CHF = congestive heart failure, CI = confidence interval, MBP = mean blood pressure, ORs = odds ratios, WBC = white blood cell.

### 3.3. Subgroup analysis

**Figure 3.**
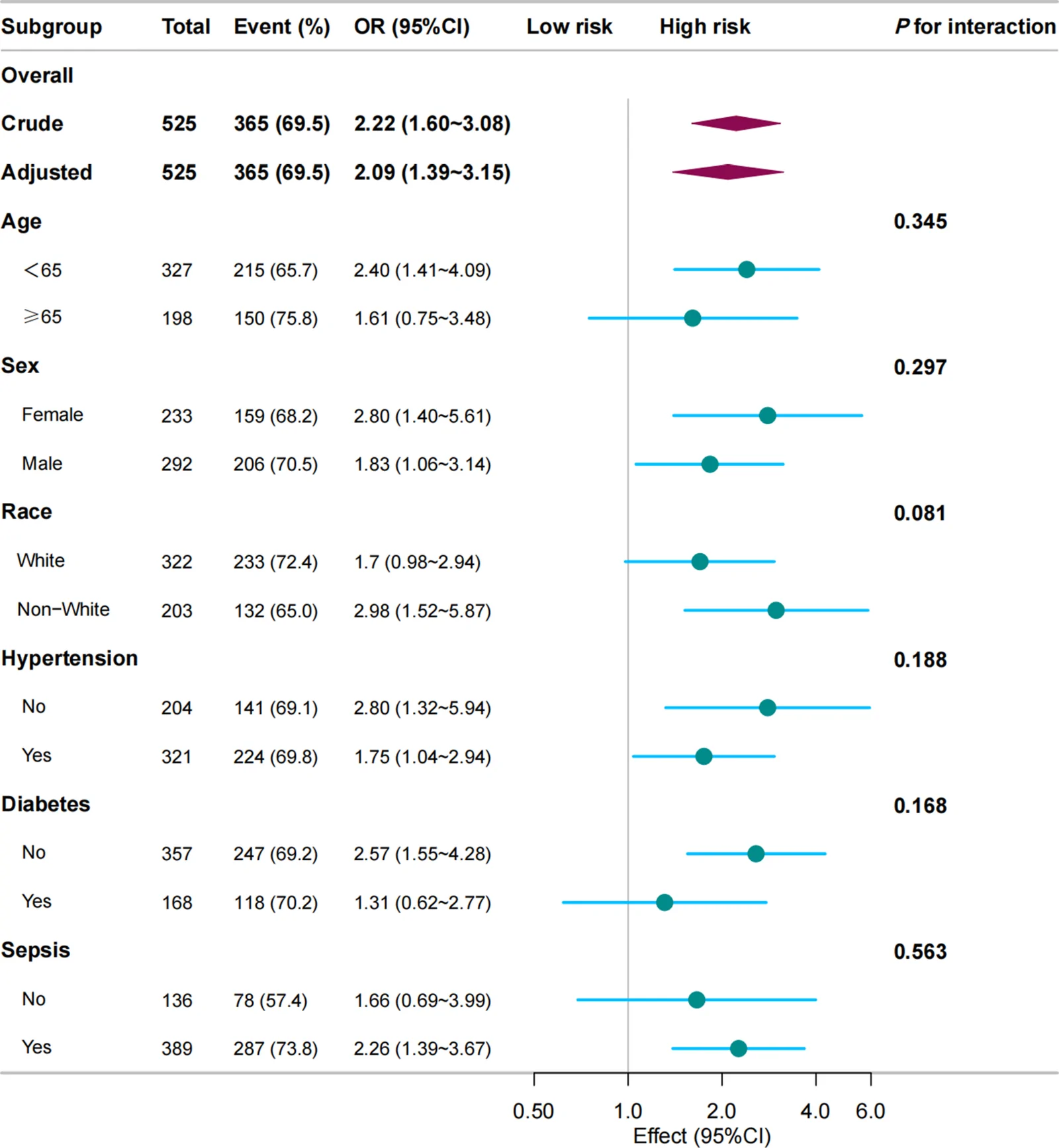
Forest plot for the association of ALBI score with AKI among different subgroups of critically ill patients with AP. Adjusted for model III, including age, sex, race, temperature, MBP, WBC, hemoglobin, platelet, AG, hypertension, diabetes, CHF, creatinine, BUN, sepsis, and vasopressors. AG = anion gap, AKI = acute kidney injury, ALBI = albumin–bilirubin, AP = acute pancreatitis, BUN = blood urea nitrogen, CHF = congestive heart failure, CI = confidence interval, MBP = mean blood pressure, OR = odds ratio, WBC = white blood cell.

Stratified and interaction analyses were performed by age, sex, race, hypertension, diabetes, and sepsis to evaluate whether the correlation between ALBI score and AKI was stable across different subgroups. As shown in the forest plot in Figure 3, there was a stable relationship between ALBI score and AKI across different subgroups. In addition, there was no significant interaction between ALBI score and each subgroup (*P* for interaction > 0.05).

### 3.4. Mediation analysis

**Figure 4.**
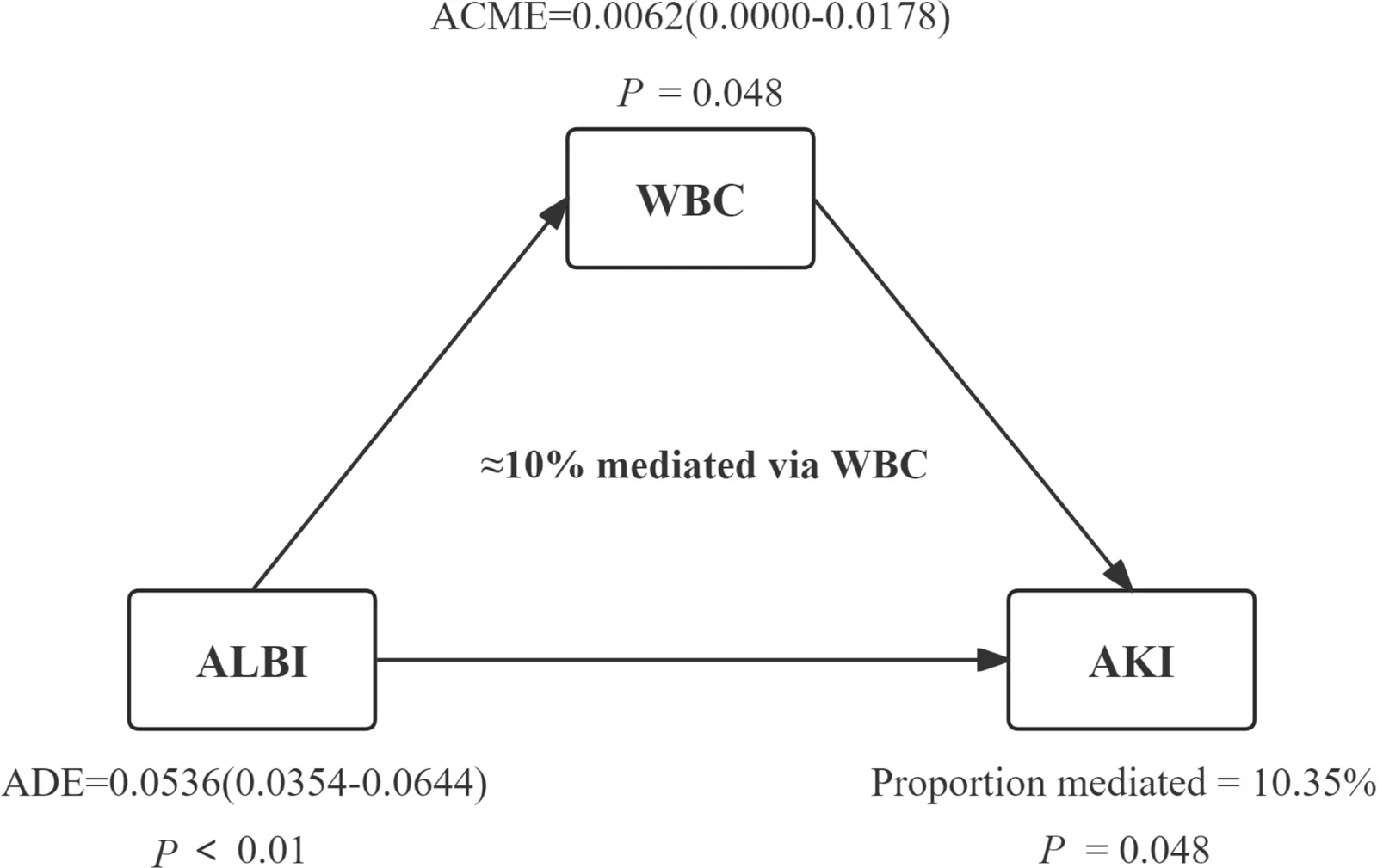
Mediation analysis for the impact of ALBI score on AKI: the mediating effects of WBC. Adjusted for age, sex, race, temperature, MBP, hemoglobin, platelet, creatinine, BUN, AG, hypertension, diabetes, and CHF. ACME = average causal mediation effect, ADE = average direct effect, AG = anion gap, AKI = acute kidney injury, ALBI = albumin–bilirubin, AP = acute pancreatitis, BUN = blood urea nitrogen, CHF = congestive heart failure, MBP = mean blood pressure, WBC = white blood cell.

Mediation analysis indicated that systemic inflammation, as represented by WBC count, accounted for a modest mediated proportion (≈10.35%, *P* = .048) of the total association between ALBI score and AKI (Fig. 4).

### 3.5. Sensitivity analysis

To validate the robustness of primary outcomes, we performed sensitivity analyses. Excluding creatinine, BUN, sepsis, and vasopressor use from model III, the results in model IV remained consistent and robust (Table S1, Supplemental Digital Content 1). Similar findings were observed in subgroup analyses, supporting the stability of the main results (Fig. S1, Supplemental Digital Content 2). The association between ALBI score and AKI remained robust when excluding patients with malignancy, liver failure, and end-stage renal disease (Table S2, Supplemental Digital Content 3). The *E*-value of this cohort was 2.34 to 2.45, which revealed our results demonstrate notable resilience to unmeasured confounding effects.

## 4. Discussion

This observational study highlights the independent association between the ALBI score and the development of AKI in critically ill patients with AP. We demonstrate a robust dose–response relationship between the ALBI score and AKI after adjusting for multiple confounders. These findings suggest that the ALBI score, which can be derived from routine laboratory tests, may serve as an accessible clinical tool for early identification of patients at high risk for renal complications in AP. Given that AKI is a well-established predictor of multi-organ failure and mortality in AP,^[12]^ early identification through the ALBI score may facilitate timely intervention, potentially improving patient outcomes.

The incidence of AKI in this cohort was higher than that reported in many noncritical AP cohorts. This discrepancy can be attributed to our use of the full KDIGO criteria for AKI diagnosis, including both Scr and urine output thresholds, as well as the fact that our study population consisted of critically ill patients admitted to the ICU, with a median SOFA score of 6.6, a high prevalence of sepsis, and frequent use of vasopressors. Our results align with previous studies that have identified several risk factors for AKI in AP, such as advanced age, elevated WBC, elevated creatinine, elevated bilirubin levels, persistent hypotension, hypoalbuminemia, sepsis, and vasopressor use.^[13–16]^ Although these variables are well-established predictors, our study is the first to explore the association between the ALBI score and AKI in this patient population. The significant association, even after adjusting for comorbidities and hemodynamic status, underscores its potential as a valuable risk stratification tool.

The pathophysiological mechanisms underlying the ALBI-AKI association likely involve both systemic inflammation and oxidative stress. The ALBI score incorporates serum albumin, which reflects nutritional status and systemic capillary leak,^[17]^ and bilirubin, which can contribute to renal injury when elevated in critical illness.^[18]^ These dual pathophysiological mechanisms, including hypoproteinemia-induced endothelial vulnerability and hyperbilirubinemia-driven oxidative stress, combine to significantly correlate with AKI development in critically ill patients with AP.^[13,19]^ In addition, our mediation analysis revealed that WBC partially mediates this relationship, explaining approximately 10% of the association between the ALBI score and AKI. While inflammation plays a role, other mechanisms, such as microcirculatory dysfunction or direct bile acid toxicity, may also contribute to the observed association. Although these findings support a potential role in inflammatory pathways, causal interpretations cannot be drawn from the present observational data.

However, as an observational study, our findings should be interpreted with caution. We emphasize that these results reflect associations rather than causal relationships. Further prospective studies are needed to establish the role of the ALBI score in guiding treatment strategies. In clinical practice, the ALBI score offers a simple, cost-effective tool for early identification of patients at elevated risk for renal complications. Nevertheless, prospective trials are needed to determine the utility of the ALBI score for clinical decision-making and to explore its potential role in guiding specific interventions.

Several limitations must be acknowledged. First, as a single-center retrospective study, selection bias may have influenced the results. Second, although *E*-value analysis suggests that unmeasured confounders would need to be substantial to explain the observed association, residual confounding remains a possibility. Clinical factors such as fluid resuscitation strategies and infection severity, which were not fully accounted for, may contribute to residual bias. In addition, AKI diagnosis was based on urine output as part of the KDIGO criteria, which could introduce misclassification bias. Third, the use of a single ALBI score at ICU admission, without considering its dynamic changes over time, represents another limitation of our study. Finally, the lack of an external validation cohort restricts the generalizability of our findings. Future multicenter prospective studies are needed to validate these results and assess the dynamic predictive value of the ALBI score.

## 5. Conclusion

Upon admission, a higher ALBI score is independently associated with AKI in ICU patients with AP. These results underscore the potential of the ALBI score as a valuable tool for early risk stratification in critically ill patients with AP. Multicenter studies with larger sample sizes are needed to validate these findings.

## Acknowledgments

The authors thank the creators and maintainers of the MIMIC-IV database for providing access to the de-identified clinical data used in this study.

## Author contributions

**Conceptualization:** Yin-ying Wang, Hui-bo Wang.

**Data curation:** Yin-ying Wang, Hui-bo Wang.

**Formal analysis:** Yin-ying Wang, Hui-bo Wang.

**Methodology:** Yin-ying Wang, Hui-bo Wang.

**Writing – original draft:** Yin-ying Wang, Hui-bo Wang.

**Writing – review & editing:** Yin-ying Wang, Hui-bo Wang.






