# Supplementary material for: Risk of acute kidney injury associated with the albumin–bilirubin score in ICU patients with acute pancreatitis: A cohort study
Source: Medicine (Baltimore). 2026 Sep 11;105(37):e50677. doi: 10.1097/MD.0000000000050677 (PMC13574421; doi:10.1097/MD.0000000000050677)
Supplement: Supplementary file 1 [file medi-105-e50677-s001.docx]

Supplementary Tab**le 1 Multivariate Logistic regression analysis of AKI among critically ill patients with AP based on the ALBI score (continuous/binary/tertiles).**

| **Variable** | **Model I** | | **Model II** | | **Model III** | | **Model IV** | |
| --- | --- | --- | --- | --- | --- | --- | --- | --- |
|  | **OR (95%CI)** | ***P* value** | **OR (95%CI)** | ***P* value** | **OR (95%CI)** | ***P* value** | **OR (95%CI)** | ***P* value** |
| Continuous | 2.22 (1.60~3.08) | <0.001 | 2.23 (1.60~3.09) | <0.001 | 2.09 (1.39~3.15) | <0.001 | 2.39 (1.61~3.55) | <0.001 |
| Binary |  |  |  |  |  |  |  |  |
| Non-Grade 3 | 1(Ref) |  | 1(Ref) |  | 1(Ref) |  | 1(Ref) |  |
| Grade 3 | 2.19 (1.46~3.28) | <0.001 | 2.29 (1.52~3.44) | <0.001 | 1.98 (1.24~3.17) | 0.004 | 2.28 (1.44~3.6) | <0.001 |
| Tertiles |  |  |  |  |  |  |  |  |
| T1 | 1(Ref) |  | 1(Ref) |  | 1(Ref) |  | 1(Ref) |  |
| T2 | 1.64 (1.05~2.54) | 0.029 | 1.58 (1.01~2.47) | 0.043 | 1.71 (1.04~2.81) | 0.034 | 1.83 (1.12~2.99) | 0.016 |
| T3 | 2.64 (1.65~4.24) | <0.001 | 2.67 (1.66~4.29) | <0.001 | 2.18 (1.24~3.84) | 0.007 | 2.64 (1.53~4.57) | <0.001 |
| *P* for trend |  | <0.001 |  | <0.001 |  | 0.005 |  | <0.001 |

Notes:

Model I：Unadjusted;

Model II：Adjusted for age, sex, race;

Model III：Adjusted for age, sex, race, temperature, MBP, WBC, hemoglobin, platelet, AG, hypertension, diabetes, CHF, creatinine, BUN, sepsis, and vasopressors;

Model IV：Adjusted for age, sex, race, temperature, MBP, WBC, hemoglobin, platelet, AG, hypertension, diabetes, and CHF.

Abbreviations: AG, Anion gap; AKI, Acute kidney injury; ALBI, Albumin-Bilirubin; AP, Acute pancreatitis; BUN, Blood urea nitrogen; CHF, Congestive heart failure; CI, Confidence interval; MBP, Mean blood pressure; ORs, Odds ratios; WBC, White blood cell.
