# Supplementary material for: Risk of acute kidney injury associated with the albumin–bilirubin score in ICU patients with acute pancreatitis: A cohort study
Source: Medicine (Baltimore). 2026 Sep 11;105(37):e50677. doi: 10.1097/MD.0000000000050677 (PMC13574421; doi:10.1097/MD.0000000000050677)
Supplement: Supplementary file 2 [file medi-105-e50677-s003.docx]

**Supplementary Table 2. Sensitivity analysis of the association between the ALBI score (continuous/binary/tertiles) and AKI in critically ill patients with AP, excluding those with malignancy, liver failure, and end-stage renal disease.**

| **Variable** | **Model I** | | **Model II** | | **Model III** | | **Model IV** | |
| --- | --- | --- | --- | --- | --- | --- | --- | --- |
|  | **OR (95%CI)** | ***P* value** | **OR (95%CI)** | ***P* value** | **OR (95%CI)** | ***P* value** | **OR (95%CI)** | ***P* value** |
| Continuous | 2.14 (1.5~3.06) | <0.001 | 2.15 (1.50~3.08) | <0.001 | 2.14 (1.35~3.38) | 0.001 | 2.49 (1.61~3.84) | <0.001 |
| Binary |  |  |  |  |  |  |  |  |
| Non-Grade 3 | 1(Ref) |  | 1(Ref) |  | 1(Ref) |  | 1(Ref) |  |
| Grade 3 | 2.03 (1.32~3.12) | 0.001 | 2.13 (1.38~3.30) | 0.001 | 1.91 (1.15~3.16) | 0.012 | 2.32 (1.43~3.79) | 0.001 |
| Tertiles |  |  |  |  |  |  |  |  |
| T1 | 1(Ref) |  | 1(Ref) |  | 1(Ref) |  | 1(Ref) |  |
| T2 | 1.99 (1.23~3.21) | 0.005 | 1.94 (1.19~3.15) | 0.007 | 2.28 (1.33~3.91) | 0.003 | 2.28 (1.35~3.86) | 0.002 |
| T3 | 2.81 (1.70~4.65) | <0.001 | 2.88 (1.73~4.78) | <0.001 | 2.61 (1.42~4.78) | 0.002 | 2.6 (1.46~4.62) | 0.001 |
| *P* for trend |  | <0.001 |  | <0.001 |  | 0.001 |  | 0.001 |

Notes:

Model I：Unadjusted;

Model II：Adjusted for age, sex, race;

Model III：Adjusted for age, sex, race, temperature, MBP, WBC, hemoglobin, platelet, AG, hypertension, diabetes, CHF, creatinine, BUN, sepsis, and vasopressors;

Model IV：Adjusted for age, sex, race, temperature, MBP, WBC, hemoglobin, platelet, AG, hypertension, diabetes, and CHF.

Abbreviations: AG, Anion gap; AKI, Acute kidney injury; ALBI, Albumin-Bilirubin; AP, Acute pancreatitis; BUN, Blood urea nitrogen; CHF, Congestive heart failure; CI, Confidence interval; MBP, Mean blood pressure; ORs, Odds ratios; WBC, White blood cell.
